# The survival impact of palliative chemotherapy dose modifications on metastatic colon cancer

**DOI:** 10.1186/s12885-022-09831-7

**Published:** 2022-07-04

**Authors:** Mohd Naqib Zainal Abidin, Marhanis Salihah Omar, Farida Islahudin, Noraida Mohamed Shah

**Affiliations:** 1 Centre of Quality Management of Medicines, Faculty of Pharmacy, Universiti Kebangsaan, Bangi, Malaysia; 2 National Cancer Institute, Ministry of Health, Putrajaya, Malaysia

**Keywords:** Relative dose intensity, Dose reduction, Dose delays, Dose modifications, Metastatic colorectal Cancer, Palliative chemotherapy, Oxaliplatin, FOLFOX, Survival

## Abstract

**Background:**

An uninterrupted dose of oxaliplatin-based cytotoxic therapy is an essential component in the standard treatment regimen of metastatic colon cancer (mCC). Data on the impacts of dose intensity reduction on the palliative treatment for patients with mCC remain scarce. Hence, this study aimed to investigate the impact of palliative chemotherapy dose modifications (DM) on the survival of patients with mCC.

**Methods:**

Patients with stage IV colon cancer who received first-line palliative FOLFOX regimen chemotherapy between 2014 until 2018 in the Oncology Department of the National Cancer Institute were conveniently sampled retrospectively to analyse the treatment efficacy. The cumulative dose and duration of chemotherapy received by the patients were summarised as relative dose intensity (RDI) and stratified as High RDI (RDI ≥ 70%) or Low RDI (RDI < 70%). Progression-free survival (PFS) and 2-year overall survival (OS) between the two groups were analysed using Kaplan-Meier survival analysis and Cox proportional hazards models.

**Results:**

Out of the 414 patients identified, 95 patients with mCC were eligible and included in the final analysis. About half of the patients (*n* = 47) completed the 12-cycle chemotherapy regimen and one patient received the complete (100%) RDI. The overall median RDI was 68.7%. The Low RDI group (*n* = 49) had a 1.5 times higher mortality risk than the High RDI group [OS, Hazard Ratio (HR) = 1.5, 95% Cl: 1.19–1.82] with a significant median OS difference (9.1 vs. 16.0 months, *p* <  0.01). Furthermore, patients with lower dose intensity showed double the risk of disease progression (PFS, HR = 2.0, 95% CI: 1.23–3.13) with a significant difference of 4.5 months of median PFS (*p* <  0.01). Gender and RDI were the independent prognostic factors of both OS and PFS.

**Conclusion:**

Reduction in the dose intensity of palliative chemotherapy may adversely affect both disease progression and overall survival among mCC patients.

## Background

The treatment of metastatic colon cancer (mCC) is mostly palliative in nature. Complications arising from the late disease stage and the associated treatment procedures often lead to a higher treatment cost and lower quality of life (QoL) [1]. For mCC patients, the objectives of treatment focus on prolongation of survival, symptom control, and improvement or maintenance of the QoL. As the mainstay of treatment in mCC, chemotherapy can be burdensome for certain patients as it is associated with a range of adverse effects that can cause psychological distress, financial difficulties, and extended hospitalisation [2]. Chemotherapy toxicity may further decline patient’s QoL and increase the treatment cost to an extent that exceeds the cost of supportive care [3]. Conversely, if the chemotherapy improves the symptoms and QoL, it may lessen patients’ needs and subsequent dependency on supportive care. Furthermore, palliative chemotherapy does have a positive effect in survival compared to best supportive care in patients with incurable cancers [4].

Systemic combination chemotherapy is the main treatment modality of mCC. About two decades ago, two new active agents for the treatment of mCC were introduced, namely oxaliplatin and irinotecan. Their concurrent use with fluorouracil (5-FU) substantially improved all parameters of treatment efficacy [5, 6]. Subsequently, the incorporation of monoclonal antibodies such as vascular endothelial growth factor and endothelial growth factor receptor as adjunct agents also improved the survival outcomes of mCC. Nevertheless, the combination of irinotecan or oxaliplatin with fluoropyrimidine therapy remains the main chemotherapeutic agent for patients with mCC [5–8]. Even though there is a fair amount of published literature on palliative chemotherapy in mCC, the actual effectiveness of this intervention remains uncertain. Furthermore, certain populations such as Asians, the elderly, and patients with metastatic disease are often underrepresented in clinical trials [3].

Based on previous work, mCC patients have a median survival of 6–9 months from the diagnosis, during which their symptoms may deteriorate, leading to a poorer QoL [9]. With cytotoxic therapy, the median overall survival (OS) was extended by approximately 4 months to 12 months, and the progression-free survival (PFS) was also prolonged to 6 months [3]. On the other hand, some published studies highlighted non-significant survival benefits from palliative chemotherapy, some even with a potential increase in mortality [10–12]. In Malaysia, 5-FU with a combination of oxaliplatin (FOLFOX) or irinotecan (FOLFIRI) is the first-line palliative chemotherapy regimen and the standard of care [13]. Despite having similar efficacy, FOLFOX is more commonly prescribed than FOLFIRI due to the better overall toxicity profile. FOLFOX has lower rates of nausea, vomiting, stomatitis, and alopecia, thus leading to significantly better patient acceptance and QoL. In comparison, FOLFIRI is usually reserved for patients with pre-existing neuropathy as oxaliplatin may worsen neuropathy [14].

Chemotherapy-induced toxicity is a common adverse effect in the management of cancer. Dose reduction and delays are frequently used to ameliorate chemotherapy-induced toxicity. Most oncologists try to minimise myelosuppression either by decreasing the chemotherapy dose or extending the chemotherapy intervals. However, such strategies might be employed at the expense of survival benefits and disease control, as suggested by previous meta-analyses [15, 16]. The delivery of full dose chemotherapy is imperative in patients with potentially curable malignancies, hence its reduction may increase the risk for recurrence and death [17]. Despite so, delaying or dose-reducing chemotherapy continues to be practised in clinical research and practice settings to prevent myelotoxicity [18]. Older age, extended hospital stay, ICU admission, poor performance status, and comorbidities have been established as strong predictors of reduction in chemotherapy dose intensity. Worst still, most of these factors are inexorable [19, 20].

The Hryniuk model is the most widely used calculation method for relative dose intensity (RDI) to ensure patient’s tolerability to the chemotherapy and adherence to protocol [21]. In metastatic solid tumour disease, an increased survival rate was observed among patients who received 70% or more of RDI. Conversely, the mortality rate curves were as dismal as untreated populations when this threshold RDI was not administered. Therefore, the maintenance of full-dose chemotherapy on a planned schedule is vital to improve patient outcomes [22]. The administration of full chemotherapy dose intensity has been shown to improve clinical outcomes in various cancers, including colon cancer. Nevertheless, RDI reduction effects on the survival outcomes among the metastatic cancer population remain inconclusive [23]. Therefore, there is a lack of evidence on the effect of maintaining full chemotherapy dose intensity on the survival of patients with advanced solid tumours [24].

Even though the practice of chemotherapy dose modification is prevalent in the clinical setting, the impacts of this practice on the survival of mCC patients are still under-researched. Thus, it is challenging to achieve an optimal balance between the risk of adverse effects and survival benefits in this fragile population [25, 26]. Therefore, this study aimed to determine the prevalence of chemotherapy dose modification and its effects on the survival of mCC patients in Malaysia. The study findings can assist healthcare providers to make well-informed decisions in personalising the dose of palliative chemotherapy during end-of-life care.

## Methods

### Study design

This was a retrospective, observational cohort study conducted in the adult wards at the Radiotherapy and Oncology Department, National Cancer Institute (IKN, *Institut Kanser Negara*), Malaysia. All data were extracted from the hospital electronic medical records (EMR). The eligibility of patients was assessed via patients’ medical records and clinical data from the EMR.

### Study population

The target population for this study was all patients who received first-line palliative FOLFOX chemotherapy regimens in IKN. All patients included in the analysis were treated with FOLFOX-4 or FOLFOX-6 therapy regimes, as recommended by the MOH Cancer Systemic Therapy Protocol 2016 [13]. All eligible patients who had been prescribed palliative FOLFOX during the data collection period were conveniently sampled into this study. They were followed up for 2 years after the initiation of palliative chemotherapy or until death, whichever came first.

In this study, chemotherapy-naïve patients with histologically confirmed adenocarcinoma of the colon in stage 4 who received FOLFOX chemotherapy regimens were included. However, the exclusion criteria were i. incomplete medical records on treatment or follow-up, ii. other histology such as neuroendocrine tumours, iii. Concurrent or history of other malignancies, iv. recurrent mCC, v. concomitant use of biologic agents during the first-line chemotherapy, vi. received less than 4 cycles of chemotherapy, and vii. Pregnant or lactating women.

### Data collection

The data collection was conducted from March 2021 to July 2021. All patients who received chemotherapy from January 2014 until December 2018 were followed up from the beginning of the first cycle of chemotherapy for 2 years, or until death or loss to follow-up, whichever that came first.

### Indicators of chemotherapy delivery

This study utilised the Relative Dose Intensity (RDI) to review the total dose of chemotherapy received as compared to the recommended clinical practice guideline. RDI was described as the proportion of total delivered dose divided by the actual duration of therapy to the standard dose divided by the planned duration of therapy, expressed as a percentage. Hence, RDI can indicate the effects of both dose reduction and dose delay [21]. As an indicator of chemotherapy dose reduction, ‘dose index (DI)’ was defined as the ratio of the actual administered total dose to the planned total dose. As for time delay, ‘time index (TI)’ was defined as the ratio of the scheduled duration to the actual duration of therapy. The planned duration was calculated by multiplying the planned duration of 1 cycle by the actual treatment cycles whereas RDI was computed by multiplying DI by TI [27]. The calculation for RDI, DI, and TI is shown below.

$$\cdotp \mathrm{Relative}\ \mathrm{dose}\ \mathrm{Intensity}\left(\mathrm{RDI}\right)\left(\%\right)=\frac{\mathrm{Delivered}\ {\mathrm{dose}\ \mathrm{intensity}}^{\ast 1}\left(\frac{\mathrm{mg}}{\mathrm{week}}\right)}{\mathrm{Standard}\ {\mathrm{dose}\ \mathrm{intensity}}^{\ast 2}\left(\frac{\mathrm{mg}}{\mathrm{week}}\right)\ }\times100$$

$${}^{\ast}\mathbf{1}\ \mathrm{Delivered}\ \mathrm{dose}\ \mathrm{Intensity}\left(\frac{\mathrm{mg}}{\mathrm{week}}\right)=\frac{\mathrm{Total}\ \mathrm{delivered}\ \mathrm{dose}\left(\mathrm{mg}\right)}{\mathrm{Actual}\ \mathrm{duration}\ \mathrm{of}\ \mathrm{theraphy}\ \left(\mathrm{week}\right)}$$

$${}^{\ast}\mathbf{2}\ \mathrm{Standard}\ \mathrm{dose}\ \mathrm{Intensity}\left(\frac{\mathrm{mg}}{\mathrm{week}}\right)=\frac{\mathrm{Total}\ \mathrm{standard}\ \mathrm{dose}\ \left(\mathrm{mg}\right)}{\begin{array}{l}\mathrm{Planned}\ \mathrm{duration}\ \mathrm{of}\ \mathrm{theraphy}\ \left(\mathrm{week}\right)\\ {}\cdotp \mathrm{Dose}\ \mathrm{index}\ \left(\mathrm{DI}\right)=\frac{\mathrm{Total}\ \mathrm{delivered}\ \mathrm{dose}\ \left(\mathrm{mg}\right)}{\mathrm{Total}\ \mathrm{planned}\ \mathrm{dose}\ \left(\mathrm{mg}\right)}\\ {}\cdotp \mathrm{Time}\ \mathrm{index}\ \left(\mathrm{TI}\right)=\frac{\mathrm{Planned}\ \mathrm{duration}\ \mathrm{of}\ \mathrm{theraphy}\ \left(\mathrm{week}\right)}{\mathrm{Actual}\ \mathrm{duration}\ \mathrm{of}\ \mathrm{theraphy}\ \left(\mathrm{week}\right)}\end{array}}$$

### Sample size

The estimated number of mCC patients who received first-line chemotherapy in Malaysia was 1439 patients in a 5-year period [28–30]. Sample size estimation was computed using the population proportion formula [31]. The margin of error was set at 5% with confidence intervals of 95%. The calculated sample size was 44 per arm. Hence, at least 88 patients were required to produce clinically meaningful results. These patients would be categorised as High RDI (RDI ≥ 70%) or Low RDI (RDI < 70%) to assess the correlation between RDI and survival outcomes [22, 24, 32].

### Survival endpoints

The endpoints of this study included the overall survival (OS) and progression-free survival (PFS). OS referred to the duration between the date of starting first-line palliative chemotherapy and date of death, with those alive censored at the last known follow-up (2 years). Next, PFS was the period between the date of starting first-line palliative chemotherapy and when tumour progression was detected. Patients with no recorded progression were censored at the date of their last known follow-up [33].

### Statistical analysis

All the data were pooled and analysed using Statistical Package for the Social Sciences (SPSS) Statistic version 26.0 (IBM Corp., Armonk, New York). Descriptive statistic was used to analyse demographical data and the reasons for dose modifications. Continuous parametric data such as age were presented as mean and standard deviation or median and interquartile range depending on the normality distribution. Kolmogorov-Smirnov equation was used to test the normality of all the continuous variables. Categorical data such as age group, gender, race, Charlson’s comorbidity score, primary tumour location, TNM status, and ECOG performance status were expressed as absolute frequencies and relative percentages. Patient characteristics were compared using t-tests for normally distributed continuous data and non-parametric Mann-Whitney or parametric Chi-square tests for categorical variables.

As abovementioned, the RDI for eligible patients was calculated using the RDI formula. The two components of RDI, DI, and TI that reflect the dose reduction and time delay respectively were also calculated. Survival analysis for both RDI cohorts was performed using Kaplan-Meier survival analysis. The two-sided Log Rank test in Kaplan-Meier analysis was used to test for statistical significance between the two cohorts (High RDI vs Low RDI), where the level of significance was set as 0.05. The follow-up period and survival times were censored using December 31st, 2020 as the cut-off date.

Furthermore, univariate and multivariate regression analyses with Cox proportional hazard models were utilised to determine the influence of covariates on PFS and OS. In the multivariate analysis, the hazard ratio (HR) was adjusted for age, gender, staging, Charlson’s comorbidity score, surgery status, and further chemotherapy, i.e. the number of treatment lines and regimens. A two-sided *p*-value of 0.05 was taken as statistical significance. The corresponding 95% confidence intervals (CI) of the HRs were calculated and considered as statistically significant when the CI excluded 1.0.

### Research ethical approval

Ethics approval was acquired from the Universiti Kebangsaan Malaysia Research Ethics Committee (UKM PPI/111/8/ JEP-2021-055) and the Medical Research and Ethics Committee Ministry of Health Malaysia (NMRR-20-3107-57,372) before the commencement of the study.

## Results

A total of 414 patients underwent chemotherapy regimens between the study period. Of these patients, 319 patients were excluded due to i. not chemotherapy-naïve (*n* = 105), ii. disease recurrence (*n* = 71), iii. Received prior adjuvant chemotherapy (*n* = 59), iv. received three or fewer cycles of chemotherapy (*n* = 18), v. had a combination treatment with a biological agent (*n* = 11), and vi. other unmet inclusion criteria (*n* = 47). None of these patients received granulocyte colony-stimulating factors throughout the chemotherapy duration. In addition, all of them received FOLFIRI as the second-line chemotherapy.

Based on the RDI, the patients were dichotomised into two cohorts; i.e. High RDI (*n* = 46) and Low RDI (*n* = 49), based on the RDI 70% cut-off. The baseline demographic and clinical characteristics for all patients, as well as after dichotomisation based on the RDI received, are summarised in Table 1. Overall, most of the patients were male (*n* = 55, 57.9%), Malay (n = 55, 57.9%), with a median (interquartile range) age of 60 (31–74) years. More than half of the patients recorded a Charlson Morbidity Score of 6 (*n* = 51, 53.7%), as the presence of metastasis would have contributed a score of 6. The depth of tumour invasion was reflected by the TNM staging system, whereby the most common T-values and N-values were T4 (36.8%) and N2 (38.9%) respectively. In addition, slightly more than half (*n* = 49, 51.6%) of them had an ECOG performance score of 1. A significant proportion of the patients also had emergency oncological surgery prior to first-line chemotherapy (*n* = 74, 77.9%). The majority of patients recorded only one metastasis site (*n* = 56, 58.9%). Approximately half of them proceeded with the FOLFIRI chemotherapy regime after experiencing treatment failure with FOLFOX (*n* = 50, 52.6%). There were no significant differences between the High and Low RDI groups for all the baseline demographic characteristics.

**Table 1:** Patients’ baseline demographics and clinical characteristics

Characteristics (%)	All patients (*n* = 95)	High RDI (*n* = 46)	Low RDI (*n* = 49)	p-value
Age				
Median [range]	60 [31–74]	59 [31–74]	60 [39–71]	0.900
< 65	71 (74.7)	34 (73.9)	37 (75.5)	0.522
≥ 65	24 (25.3)	12 (26.1)	12 (24.5)	
Gender				
Female	40 (42.1)	18 (39.1)	22 (44.9)	0.359
Male	55 (57.9)	28 (60.9)	27 (55.1)	
Race				
Malay	55 (57.9)	28 (60.9)	27 (55.1)	0.667
Chinese	32 (33.7)	14 (30.4)	18 (36.7)	
Indian	7 (7.4)	4 (8.7)	3 (6.1)	
Other	1 (1.0)	0	1 (2.1)	
Charlson’s Comorbidity Score				
6	51 (53.7)	25 (54.3)	26 (53)	0.777
7	33 (34.7)	17 (37.0)	16 (32.7)	
8	7 (7.4)	3 (6.5)	4 (8.2)	
9	4 (4.2)	1 (2.2)	3 (6.1)	
Primary Tumour location				
Colon	40 (42.1)	18 (39.1)	22 (44.9)	0.359
Rectum	55 (57.9)	28 (60.9)	27 (55.1)	
TNM - T Status				
T2	5 (5.3)	1 (2.2)	4 (8.2)	0.612
T3	42 (44.2)	21 (45.7)	21 (42.9)	
T4	35 (36.8)	18 (39.1)	17 (34.6)	
Tx	13 (13.7)	6 (13.0)	7 (14.3)	
TNM - N Status				
N0	8 (8.5)	5 (10.8)	3 (6.1)	0.692
N1	36 (37.9)	17 (37)	19 (38.8)	
N2	37 (38.9)	18 (39.1)	19 (38.8)	
N3	1 (1.0)	1 (2.2)	0	
Nx	13 (13.7)	5 (10.9)	8 (16.3)	
ECOG Performance Score				
0	30 (31.6)	17 (37.0)	13 (26.5)	0.258
1	49 (51.6)	24 (52.2)	25 (51.0)	
2	16 (16.8)	5 (10.8)	11 (22.5)	
Oncological Surgery Status				
No	21 (22.1)	9 (19.6)	12 (24.5)	0.371
Yes	74 (77.9)	37 (80.4)	37 (75.5)	
No. of metastasis site				
1	56 (58.9)	28 (60.9)	28 (57.2)	0.915
2	33 (34.7)	15 (32.6)	18 (36.7)	
≥ 3	6 (6.4)	3 (6.5)	3 (6.1)	
Therapy after FOLFOX				
Best supportive care	43 (45.3)	19 (41.3)	24 (49)	0.288
FOLFIRI	50 (52.6)	25 (54.3)	25 (51)	
Capecitabine	2 (2.1)	2 (4.4)	0	
No. of chemotherapy cycles received				
Median [range]	11 (4–12)	12 (9–12)	6 (4–12)	< 0.001

From the data, three-quarters (73.7%) of the 95 patients reported a total of 141 reasons for chemotherapy dose reductions or delays. Treatment-related reasons (*n* = 109, 77.3%) accounted for the biggest proportion, in which the majority of the reasons was associated with active infection (*n* = 33, 23.4%) that included two cases of febrile neutropenia, followed by thrombocytopenia (*n* = 17, 12.1%), worsening of general condition (*n* = 14, 9.9%), and deterioration of liver function (n = 14, 9.9%). Patient-related dose modification events (*n* = 32, 22.3%) were less frequent and included poor general condition prior to cycle 1 of chemotherapy (*n* = 12, 8.5%), followed by the need to attend to social circumstances such as family events and public holidays (*n* = 9, 6.4%), chemotherapy day clashed with other procedures (*n* = 6, 4.3%) and patient’s request (*n* = 5, 3.5%) (Table 2).

**Table 2:** Reasons for chemotherapy scheme modifications

Reasons for dose modifications	All patients (*n* = 141)	High RDI (n = 49)	Low RDI (*n* = 92)
Treatment-related reasons, n (%)			
Anaemia	3 (2.1)	–	3 (3.2)
Deterioration of kidney function	6 (4.3)	2 (4.1)	4 (4.3)
Neutropenia	7 (5.0)	–	7 (7.6)
Peripheral Neuropathy	7 (5.0)	5 (10.2)	2 (2.2)
Fatigue	8 (5.7)	3 (6.1)	5 (5.4)
Deterioration of liver function	14 (9.9)	–	14 (15.2)
Worsening of general condition	14 (9.9)	5 (10.2)	9 (9.8)
Thrombocytopenia	17 (12.1)	7 (14.3)	10 (10.9)
Infections	33 (23.4)	16 (32.7)	17 (18.5)
Total	109 (77.3)	38 (77.6)	71 (77.2)
Patient-related reasons, n (%)			
Patient request	5 (3.5)	2 (4.1)	3 (3.2)
Procedure	6 (4.3)	2 (4.1)	4 (4.3)
Social circumstances	9 (6.4)	1 (2.0)	8 (8.7)
Poor ECOG PS at Cycle 1	12 (8.5)	6 (12.2)	6 (6.5)
Total	32 (22.7)	11 (22.4)	21 (22.8)

Of all the 95 patients, only one patient received a full RDI, i.e. full recommended dose at exactly every 14 days for each of the 12 cycles. About one-fifth of the patients received at least an RDI of 90% and above (*n* = 18). Overall, the estimated median RDI for all patients was 67.7% (range 18.8–100.1). The median DI and TI were 0.82 (range 0.23–1.00) and 0.90 (range 0.59–1.02), respectively. There was a significant difference in the median RDI between the High RDI and Low RDI groups (84.3% vs 45.5%, *p* <  0.001). The same was observed for DI, whereby patients in the High RDI cohort received almost double the dose compared to the Low RDI group (0.98 vs 0.50, p <  0.001). On the other hand, there was no significant difference between the two-dose groups for TI (0.98 vs 0.50, *p* = 0.052). The overall median of the number of delayed days was 11 (range 0–88]. Table 3 summarises the RDI, DI, and TI for both groups.

**Table 3:** Relative dose intensity, dose index and time index

Clinical Characteristics, median [range]	All patients (n = 95)	High RDI (n = 46)	Low RDI (n = 49)	p-value
RDI (%)	67.7 [18.8–100.1]	84.3 [70.8–100.1]	45.5 [18.8–70.0]	< 0.0001
Dose Index, DI	0.80 [0.23–1.00]	0.98 [0.78–1.00]	0.50 [0.23–0.92]	< 0.0001
Time Index, TI	0.90 [0.59–1.02]	0.91 [0.72–1.00]	0.88 [0.59–1.02]	0.052

**Fig. 1:**
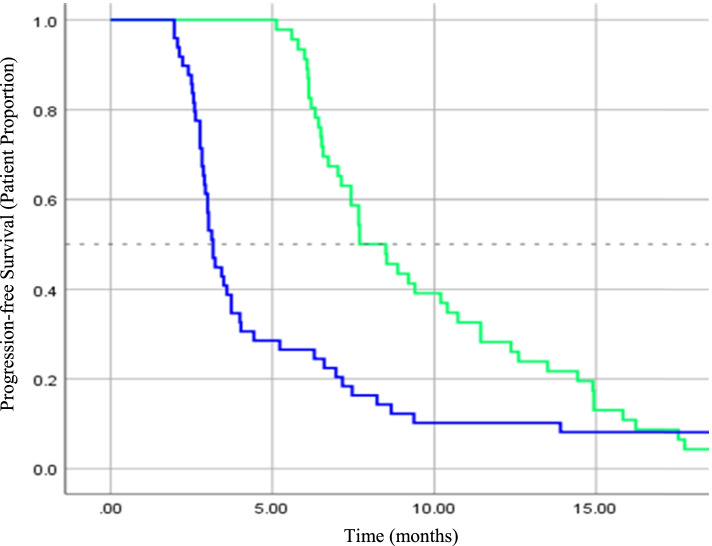
Kaplan Meier analysis of 2-year progression-free survival (PFS) in patients with High RDI (Green, n = 46) and Low RDI (Blue, n = 49). Hazard ratio = 1.96 (95% CI: 1.23–3.13), *p* < 0.01

**Fig. 2:**
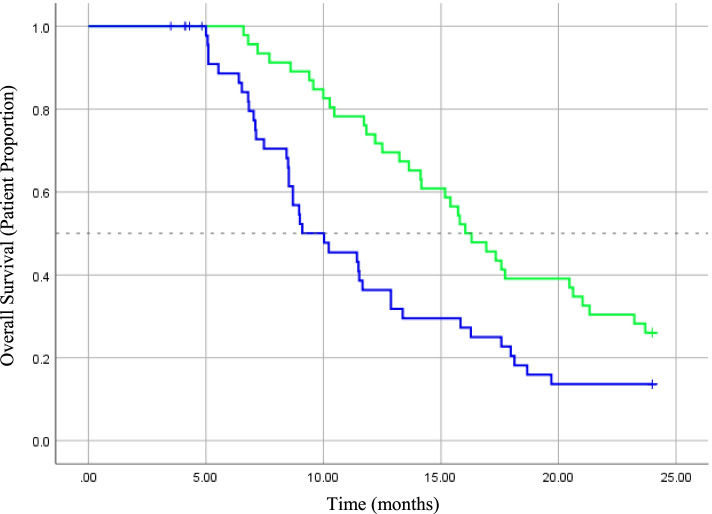
Kaplan Meier analysis of 2-year overall survival (OS) in patients with High RDI (Green, *n* = 46) and Low RDI (Blue, *n* = 49). Hazard ratio = 1.47 (95% CI: 1.19–1.82), *p* < 0.01

In terms of survival, seven (7.4%) patients were still alive at the point of analysis. Of the seven patients, four patients did not have any disease progression (i.e. stable disease) after completing the palliative chemotherapy. On a further note, both of the PFS and OS differences between the two cohorts were significant. The two-year survival for the two cohorts was 26.1% (High RDI) vs 12.2% (Low RDI). The median PFS for High RDI and Low RDI cohorts was 7.7 months (95% CI 6.3–9.1 months) and 3.2 months (95% CI 2.9–3.4 months) (Log Rank, *p* < 0.001) respectively. While for OS, the High RDI cohort showed a median of 16.0 months (95% CI 13.9–18.2 months) compared to 9.1 months (95% CI 6.-12.1 months) for the Low RDI cohort (Log Rank, *p* = 0.004). As shown in Figs. 1 and 2, the Kaplan-Meier plots outline the overall survival and disease progression over the 2-year observation period for both cohorts.

Univariate analyses showed that the RDI during FOLFOX therapy was significantly and positively associated with both survival endpoints. For PFS, the univariable Cox regression rendered HR of 1.96 (95% CI 1.23–3.13, *p* < 0.01) for the Low RDI group versus High RDI. In addition, patients who received higher dose intensity were associated with better OS than those in the Low RDI cohort (HR = 1.47, 95% CI: 1.19–1.82, *p* < 0.01). Multivariate analysis was performed to determine the predictors of RDI in both cohorts in terms of age, gender, ethnicity, Charlson’s comorbidity score, ECOG performance status, oncological surgery status, number of metastasis sites, chemotherapy sequence, and primary tumour location.

Consistent with the univariate analysis, the multivariate analyses showed a significant impact of RDI reduction on both OS and PFS. For OS, patients in the Low RDI group had three times higher risk of mortality compared to the High RDI group (HR 2.69; 95% CI 1.50–4.82, *p* < 0.01). Similarly, patients in the Low RDI group were four times more likely to develop disease progression than the High RDI group (HR 4.1; 95% CI 2.39–7.03, p < 0.01). Independent prognostic factors for these cohorts included gender, whereby male patients showed better OS (HR 0.39; 95% CI 0.20–0.72, p < 0.01) and PFS (HR 0.57; 95% CI 0.33–0.96, *p* = 0.04) after receiving FOLFOX (Table 4).

**Table 4:** Multivariate analysis of factors predictive of survival endpoints

	Overall Survival^a^		Progression-free Survival^b^	
	HR^c^ (95% CI)	p-value	HR^c^ (95% CI)	p-value
**RDI**				
High RDI	Reference		Reference	
Low RDI	2.69 (1.50–4.82)	< 0.01*	4.10 (2.39–7.03)	< 0.01*
**Age**				
< 65	Reference		Reference	
≥ 65	0.79 (0.40–1.57)	0.50	0.71 (0.38–1.35)	0.30
**Gender**				
Female	Reference		Reference	
Male	0.38 (0.20–0.72)	< 0.01*	0.57 (0.33–0.96)	0.04*
**Race**				
Malay	Reference		Reference	
Chinese	0.73 (0.08–6.63)	0.78	0.62 (0.07–5.34)	0.66
Indian	1.16 (0.13–10.65)	0.89	0.79 (0.09–7.04)	0.83
Other	0.61 (0.05–7.09)	0.70	2.25 (0.21–24.28)	0.50
**Charlson’s Comorbidity Score**				
	0.86 (0.61–1.23)	0.41	0.85 (0.60–1.20)	0.35
**ECOG Performance Score**				
0	Reference	0.10	Reference	0.57
1	0.41 (0.17–1.01)	0.05	0.65 (0.29–1.46)	0.30
2	0.68 (0.29–1.58)	0.37	0.70 (0.33–1.46)	0.34
**Oncological Surgery Status**				
No	Reference		Reference	
Yes	1.48 (0.71–3.10)	0.30	1.64 (0.87–3.09)	0.12
**No. of metastasis site**				
1	Reference		Reference	
2	0.90 (0.28–2.83)	0.85	1.04 (0.37–2.94)	0.94
≥ 3	1.15 (0.37–3.57)	0.80	1.11 (0.39–3.15)	0.84
**Chemotherapy Sequence**				
FOLFIRI	Reference		Reference	
BSC	1.75 (0.29–10.60)	0.54	1.56 (0.27–9.14)	0.62
Capecitabine	5.39 (0.88–31.73)	0.07	0.78 (0.13–4.60)	0.78
**Cancer location**				
Colon	Reference		Reference	
Rectum	0.71 (0.40–1.27)	0.25	0.91 (0.54–1.54)	0.73

^a^A HR > 1 indicates the covariate is associated with an increased risk of death from any cause, and therefore, decreased OS

^b^A HR > 1 indicates the covariate is associated with an increased risk of disease progression and therefore, decreased PFS

^c^Multivariable COX regression analysis adjusted for RDI groups, age, gender, ethnicity, Charlson’s Comorbidity Score, ECOG performance score, oncological surgery, number of metastasis sites, chemotherapy sequence and main tumour location

*p-value < 0.05 denotes statistical significance

*Abbreviations: HR* – Hazard Ratio, *CI* – Confidence Interval, *RDI* – Relative dose intensity

## Discussion

The distribution of mCC patients in this study was nationally representative of the colon cancer demographics in Malaysia based on the comparison with the descriptive statistics of colorectal cancer cases in the National Cancer Patient Registry from 2008 to 2013 [34]. The gender distribution in this study was similar when compared against the registry, in which male patients accounted for more than half of colon cancer patients. Age-wise, more than half of the population were less than 65 years old upon diagnosis of colon cancer. However, in terms of ethnicity, there was a high proportion of the Malay population in this study (57.9%) when compared to the registry data for colon cancer (42.7%) [34].

The predictive factors of cancer progression are crucial as prognostic factors to predict survival [35]. Given the scarcity of literature, this study was compared against the study by Munker et al. with a similar population [36]. Our study identified similar prognostic factors in the sampled mCC population as Munker et al. Among these, the most prominent predictors were Charlson’s comorbidity score of 6, having oncological surgery prior to first-line chemotherapy (more than 70%), and switching to FOLFIRI after FOLFOX failure (more than half).

In this study, patients above 65 years old constituted one-quarter of the total sample. Elderly age was associated with a lower rate of receiving full recommended dose treatment due to comorbidities and fear of toxicity [37]. Furthermore, they experience more physiological changes such as alterations in organ function and volume of distribution that may affect the pharmacokinetic profile of the drugs, thus resulting in a higher risk of disproportionate toxicity compared to the younger population [38]. Therefore, reduced dose intensity is occasionally necessary for very elderly, frail, and debilitated patients. These modifications may sometimes be appropriate for patients with advanced disease, especially when the therapy is focused mainly on symptom control [39]. Nevertheless, RDI was evenly distributed among the elderly and younger age groups in this study.

To date, the values of RDI ranged from 55% up to 85% and no standardised cut off point of RDI has been defined to evaluate the impact of chemotherapy dose modifications in various malignancies [23]. The cut-off point of 70% was used in this study based on the published literature. It is expected that at least 70% of the standard dose is necessary to maintain similar survival outcomes as the standard dose [22, 24, 32]. The median RDI of 67.7% in this study was lower than the median RDI of 80% reported by Nakayama et al. that retrospectively evaluated RDI data from RCT [25]. However, the study reported similar median values of DI and TI of FOLFOX in mCC patients, namely 0.97 (range 0.76–1.04) and 0.82 (range 0.55–1.00) respectively.

The study results indicated that the RDI reduction of FOLFOX chemotherapy negatively affected the survival benefit. In terms of the median values of PFS, there was a significant difference of 4.5 months (*p* < 0.001) between the High RDI and the Low RDI group. Univariate analyses showed that patients who received lower RDI were about two times more likely to develop disease progression (PFS HR = 1.96 (95% CI 1.23–3.13; *p* < 0.01)). Nakayama et al. highlighted a similar observation on the effects of RDI reduction on the PFS in mCC patients whereby the lower RDI group recorded almost three times higher risk of disease progression (HR 2.74; 95% CI 1.02–7.33, *p* = 0.04) [25]. In addition, Maindrault-Goebel et al. also showed that higher dose FOLFOX significantly improved the disease response rate and PFS compared to lower dose intensity [26]. However, in another study by Park et al., no significant difference was detected between the patients who received FOLFOX of RDI lesser than 60% [40]. The insignificant difference could be explained by the adjuvant setting in the study whereby the chemotherapy was instituted right after the primary treatment, i.e. the tumour removal surgery.

In addition, this study demonstrated that the survival rate was significantly and negatively associated with a dose reduction of the FOLFOX regime. With a significant median difference of 7 months, this study showed that the patients in the High RDI had a longer survival (OS; High RDI, 16.0 vs Low RDI, 9.1 months, *p* = 0.004). On a similar note, patients in the Low RDI cohort had an elevated risk of mortality than the High RDI patients (OS; HR = 1.47 (95% CI: 1.19–1.82), *p* < 0.01). However, Munker et al., the only other published literature that investigated the effect of FOLFOX RDI reduction among mCC patients found no effect on the OS for patients who received a lower RDI [36]. Other studies in the adjuvant settings also demonstrated similar results on OS despite a significant effect of PFS [22, 40]. This discrepancy could be explained by the inclusion of healthier and younger patients in the trials as compared to our study population. Besides, there was a higher proportion of patients who received second-line chemotherapy and biological agents in those trials, both of which could have a significant effect on the OS.

In view of the positive impact of higher RDI, the modifiable treatment- and patient-related factors in this population must be tackled. Strategies to improve RDI may include routine calculation of RDI, assessment of febrile neutropenia risk factors to maximise the use of prophylactic granulocyte colony-stimulating factor medications, enforcement of a cancellation policy, and enhanced multidisciplinary education and counselling with patients and families to ensure optimal patient outcomes [41]. Consistent with other RCTs and retrospective studies, a lower RDI was significantly associated with poorer tumour response and survival benefits in various cancer including breast cancer, lymphoma, and colon cancer. The multivariable COX regression analysis reaffirmed the finding from the univariate analysis whereby a lower RDI was associated with poorer survival endpoints. In a palliative setting, however, dose intensity reduction is sometimes necessary due to the ‘sicker’ condition of the patients that put them at higher risk of toxicities when prescribed with a standard FOLFOX dosing.

Lastly, the male gender was a significant independent risk predictor for survival in this study. A male mCC patient who received FOLFOX chemotherapy with RDI higher than 70% had half the likelihood and disease progression and mortality when compared to females. To the best of our knowledge, no other literature has reported a similar finding. In general, it is well accepted that women had a longer survival after colorectal cancer surgery as they tended to respond better to adjuvant chemotherapy [42].

Although this study performed a comprehensive analysis in a relatively homogenous population, there are several limitations. First, a confounding effect might arise as patients who remained in the treatment for extended periods would by definition, accumulate a higher RDI. In comparison, those with rapid disease progression or treatment-resistant disease would have a lower RDI. Secondly, no follow-up data on subsequent chemotherapy among those who had disease progression were collected. The therapies received after the failure of the first-line chemotherapy could have significant effects on the OS. Hence, PFS is a more reliable endpoint to describe survival benefits. There was also no data on the RAS and RAF mutations or microsatellite instability status as these were not routinely determined in our setting during the study period. Data on the location of the tumour mass were also not consistently reported in the EMR. These may have an impact on the prognosis of mCC patients.

Although this study performed a comprehensive analysis in a relatively homogenous population, there are several limitations. First, a confounding effect might arise as patients who remained in the treatment for extended periods would by definition, accumulate a higher RDI. In comparison, those with rapid disease progression or treatment-resistant disease would have a lower RDI. Secondly, no follow-up data on subsequent chemotherapy among those who had disease progression were collected. The therapies received after the failure of the first-line chemotherapy could have significant effects on the OS. Hence, PFS is a more reliable endpoint to describe survival benefits. There was also no data on the RAS and RAF mutations or microsatellite instability status as these were not routinely determined in our setting during the study period. Data on the location of the tumour mass were also not consistently reported in the EMR. These may have an impact on the prognosis of mCC patients.

Next, information on the side effects of chemotherapy was not always available in the EMR. Hence, the grading of chemotherapy adverse effects and the relationship between toxicity and RDI reduction could not be performed quantitatively to justify the dose modification. Finally, this single-centre observational study reflected the real-world evidence of individualised chemotherapy for patients. However, the results could not be generalised to all hospitals in Malaysia. Furthermore, it was not sufficient to conclusively determine the effects of RDI reduction on the survival of mCC patients as the outcomes might have been influenced by unidentified confounding factors.

For likeminded researchers, the quality of data can be improved by conducting a prospective cohort study involving multiple centres in the future. Bigger sample size and study power can enhance the generalisability of the results to a wider local population. In addition, survival prolongation is no longer the primary concern in the metastatic population. QoL is a more preferred outcome of choice as it considers the patients’ subjective feelings about their health during the treatment as they are experiencing the adverse effects to ensure a more objective endpoint awareness.

## Conclusions

In summary, the findings from this study have filled an essential void in the literature regarding the association between the effects of chemotherapy dose reduction and delay on the survival of end-stage colon cancer patients. Despite the recommendation on chemotherapy dosage, the inevitable dose and schedule disparity continues to exist in clinical practice in this fragile population. The significant risk predictors of death and disease progression in this study were female gender and RDI lower than 70%. However, the chemotherapy dosing must also take into consideration the complexities of pharmacological treatment for advanced colon cancer. Given the paucity of relevant information on RDI in the literature, this study highlighted that patients with stage IV colon cancer who received ≥70% RDI had better 2-year OS and PFS. In addition, this study offered important “real world” data that proved the effectiveness of palliative chemotherapy in the metastatic setting and also highlight the importance of administering the recommended doses of chemotherapy.

## Data Availability

The datasets generated and/or analysed during the current study are not publicly available due to data ownership by the Ministry of Health but are available from the corresponding author on reasonable request.
